# *“My young life, finished already?”:* a qualitative study of embedded social stressors and their effects on mental health of low-wage male migrant workers in Singapore

**DOI:** 10.1186/s12992-023-00946-5

**Published:** 2023-07-08

**Authors:** Aysha Farwin, Amanda Low, Natasha Howard, Huso Yi

**Affiliations:** 1 Saw Swee Hock School of Public Health, National University of Singapore and National University Health System, 12 Science Drive 2, Singapore, 117549 Singapore; 2 Department of Global Health and Development, London School of Hygiene & Tropical Medicine, London, UK

**Keywords:** Migrant workers, Social stress, Vulnerability, Mental health, Stress responses, Health inequity

## Abstract

**Background:**

Increasing evidence shows low-wage migrant workers experience a high prevalence of mental health disorders and adverse health outcomes. Significant disparities in health services usage among migrant workers create added vulnerability to health complications. However, much remains unclear about how vulnerabilities are constructed in migrant worker populations. Additionally, no studies in Singapore have attempted to critically examine the degree to which social environment and structures affect the health and wellbeing of migrant workers. Therefore, this study aimed to critically situate the socio-structural factors creating conditions of vulnerability among migrant workers using a social stress perspective.

**Methods:**

We conducted semi-structured individual and group interviews with migrant workers focused on individual life experiences, community experiences (individual and collective social capital), health (mental and physical health concerns) and stress response behaviours. We used a grounded theory approach to identify sources of stress and stress responses and uncover pathways to social vulnerabilities.

**Results:**

Findings from 21 individual and 2 group interviews revealed that migrant workers were embedded in a cycle of chronic stress driven by structural factors that were mutually reinforced by stressors arising from their social environment. Socio-structural stressors enacted as poor living, working and social conditions resulted in their negative quality-of-life appraisal. Stressors arising from being “foreign” resulted in anticipated stigma, concealment, and healthcare avoidance. These factors synergistically created a persistent mental health burden for migrant workers.

**Conclusions:**

Findings highlight the need to address the mental health burden placed on migrant workers and create avenues for migrant workers to seek psychosocial support to manage their stressors.

**Supplementary Information:**

The online version contains supplementary material available at 10.1186/s12992-023-00946-5.

## Background

Globally, an estimated 281 million migrant workers lived outside their countries of origin, comprising approximately 4.9% of the labour force of receiving countries in 2020 [1, 2]. Despite significant contributions to economic growth, migrant workers are generally excluded from essential services and protective systems in receiving countries [3, 4]. Additionally, migrant workers are predominantly employed in labour-intensive manual jobs, commonly referred to as “dirty, dangerous and difficult”, putting them at increased risk of precarious working conditions and occupational hazards [5]. Consequently, they face increased susceptibility to social and economic hardships and worsened health outcomes [6]. In particular, migrant workers are at disproportionately higher risk of suffering from poor mental health [7, 8] and several studies have shown that migrant workers experienced a greater prevalence of mental health disorders compared to native populations [9, 10]. For instance, a study with migrants in Switzerland found that 57.2% presented symptoms of at least one mental health disorder while about 45% and 36% suffered from depression and anxiety respectively [11]. Another study with migrant workers in India found 27.5% suffered from psychological distress [12]. High rates of depression and anxiety have been found among migrant workers worldwide [8, 13].

Despite significant mental health concerns, evidence shows disparities in use of health services among migrant workers, particularly mental health services [9, 14]. As non-residents, migrant workers are excluded from universal health coverage and subsidized healthcare in most receiving countries, incurring high out-of-pocket payments for healthcare [4, 15, 16]. Non-resident status often hinders migrant workers from accessing psychosocial support services that could buffer the effects of mental health issues [8, 17]. Additionally, poor health literacy, linguistic and cultural differences often hamper migrant workers’ ability to seek timely healthcare [18, 19] These barriers further exacerbate existing health inequalities and put migrant workers at a heightened risk of health adversities and poor health outcomes. While there is ample evidence indicating that migrant workers suffer from poorer mental health compared to resident populations, there is scant examination of the sources of vulnerability that predispose migrant workers to mental health disparities. Of particular interest is the idea that vulnerabilities encountered by migrant workers are differentially distributed and influenced by embedded socio-structural factors and the positions occupied by migrant workers in social structures.

### Social stress theory and health disparities

Social stress theory provides a theoretical framework to explain health disparities observed in marginalized groups [20]. This theory posits that conditions in the social environment are sources of stress for members of marginalized groups, which in turn cause negative health outcomes and maladaptive stress responses [21, 22]. According to this theory, marginalized social groups experience more pronounced stress at greater and more frequent levels due to their social position combined with general stressors. This excess exposure to stress puts marginalized individuals at heightened risk of health adversities [23]. Additionally, this theory recognizes that stressors arise from structural arrangements in which individuals are embedded and the systemic incongruence between their needs and that of the social structure [24, 25]. This incongruence creates chronic stress tied to stable social structures, resulting in marginalized individuals having to make additional efforts to cope with stressors encountered in their everyday lives.

Importantly, social stress theory emphasizes the stress-buffering resources that operate on a group level to minimize the impact of stressors on the mental health of marginalized groups [26]. Like all individuals, members of marginalized social groups use a variety of personal stress response mechanisms to withstand stressful experiences. However, as stress-buffering resources are socially distributed, this theory highlights the need to implement supportive social systems to modulate the excess stress encountered by marginalized groups and protect them from negative self-appraisal. Thus, using the social stress perspective allows us to critically examine the sources of stress associated with social structures encountered by migrant workers and identify how vulnerability is constructed in the migrant worker population.

### Study context

In 2019, it was estimated that a total of 10.1 million international migrant workers lived in four net destination countries – Brunei, Malaysia, Singapore and Thailand – in Southeast Asia [27, 28]. The island state of Singapore, in particular, is one of the largest importers of foreign labour, with total foreign workforce constituting about 25% of the total population [29]. Singapore adopts a bifurcated, two-pronged foreign labour policy in which unskilled and low-skilled workers are subject to different labour schemes compared to higher-skilled workers, who are typically better educated and in managerial positions. Under the Employment of Foreign Manpower Act (EFMA) that regulates the import of foreign labour into Singapore, unskilled and low-skilled workers (hereafter known as low-wage migrant workers) are employed under the Work Permit (WP) scheme while higher-skilled workers are employed under the Employment Pass (EP) scheme. The Work Permit scheme is a temporary foreign worker programme designed to employ low-wage migrant workers to fulfil labour shortages in Singapore [30]. As such, workers admitted under the WP scheme are subject to stricter state control and differential protections under EFMA. The higher-skilled Employment Pass holders are entitled to minimum salaries (at least $5000 per month) with employment benefits such as paid annual leave and medical insurance [31]. In addition, Employment Pass holders are also allowed to freely change jobs, bring dependents to Singapore and integrate into the community by applying for permanent residency in Singapore [30, 32].

Migrant workers, employed under the Work Permit scheme, are bound to a restrictive sponsorship system known as “kafala” that prevents them from economic and social mobility unlike their higher skilled counterparts [33, 34]. Work Permit holders are tied to the employer, job type and sector they were hired for and are prohibited from changing their jobs or job sectors freely. Work Permit passes have a limited validity of 2 years, and employers have the power to terminate passes without consequence at any time. Additionally, low-wage migrant workers are not entitled to minimum wages, paid annual leave, or medical benefits and basic salary is determined by their skill level (i.e. median monthly salary of SGD 800 [35, 36]. They are also disallowed from applying for permanent residency in Singapore or bringing their dependents to Singapore. As a result, low-wage workers are stuck in a cycle of job precarity and transience as they are prevented from establishing roots in Singapore [37].

Furthermore, while both Work Permit and Employment Pass holders are excluded from subsidized healthcare and social welfare in Singapore as foreigners, Work Permit holders face significant financial barriers to seeking healthcare due to low wages and job precarity [38, 39]. Under the EFMA, employers of Work Permit holders (i.e. low-wage migrant workers) are required to purchase and maintain medical insurance coverage of at least S$15,000 per year to cover hospitalization and day surgery costs, including hospitalization bills for conditions that are not work-related [40]. However, the mandatory medical insurance only includes coverage for inpatient admissions and day surgery and does not cover emergency services (e.g. when a worker goes to the hospital Accident and Emergency department), outpatient medical treatment, specialist medical care, or the costs of acute care needs of these workers. Although employers are responsible for the “upkeep and maintenance” of migrant workers [41], including provision of medical treatment, in practice, migrant workers bear the costs of acute care themselves, incurring high out-of-pocket costs. This serves to discourage low-wage migrant workers from seeking timely healthcare [39]. Low-wage migrant workers are also subject to pervasive surveillance and policing of their bodies. All non-domestic low-wage migrant workers are required to undergo medical examinations every 2 years to be eligible for Work Permit renewal. These medical examinations serve to screen out workers who are either in poor health and unable to contribute productively or have contracted infectious diseases such as tuberculosis, HIV, syphilis, or malaria [42]. Low-wage migrant workers who are not in satisfactory health are typically repatriated to their home countries by employers without recourse.

As of December 2022, there were 683,500 Work Permit holders in Singapore, comprised of 268,500 female migrant domestic workers and 415,000 male migrant non-domestic workers. Most male migrant non-domestic workers come from low-income countries such as Bangladesh, China, India, Myanmar, and Sri Lanka, and are predominantly employed in labour-intensive sectors such as construction, marine shipyard and processing [43–45]. Despite Singapore’s reliance on low wage labour and migrant workers being a permanent fixture of the labour force, the sentiments surrounding migrant labour have largely been negative. Studies report that migrant workers encounter discrimination and exclusion from host communities due to their identity as “foreigners” [46], alongside stressful circumstances such as family separation, job insecurity, cultural and linguistic barriers and the strains of low-wage work [47, 48]. Consequently, high prevalence of psychological distress, depressive symptoms and anxiety disorders have been reported among migrant workers in Singapore [39, 49, 50]. Mental health concerns among migrant workers became an urgent priority during the novel coronavirus disease 2019 (COVID-19) pandemic, further exacerbating anxiety and stress levels [51].

Though poor mental health has been extensively reported among migrant workers in Singapore, there is inadequate scrutiny of the embedded socio-structural factors that create stressors negatively affecting migrant workers. Additionally, critical investigation of how social environment and structures construct vulnerabilities that cause debilitating effects to the health of migrant workers is lacking. Particularly, few studies in the Singaporean context have attempted to critically examine the degree to which the social environment in which migrant workers are embedded affects them and how migrant workers in turn, navigate and negotiate these elements. To advance understanding of how social vulnerabilities are constructed among migrant workers, this study aimed to situate sources of stress contributing to adverse mental health outcomes using a social stress perspective. Identifying sources of social vulnerability among migrant workers will contribute information to needs assessments and development of interventions to reduce vulnerabilities and build psychosocial resilience.

## Methods

### Study design and research questions

We adopted an exploratory qualitative approach, using semi-structured individual and group interviews to understand the experiences, responses and interpretations of stressful experiences encountered and construct sources of vulnerability among migrant workers. This study drew on the social stress perspective to create a roadmap for situating stressors encountered by migrant workers in Singapore’s labour system. The social stress perspective allowed us to examine the mechanisms through which vulnerabilities were created in the social system in which migrant workers were embedded and examine the resulting systemic incongruence.

Our research questions were informed by literature on migrant workers in Singapore and verbal or written reports from NGO staff serving low-wage migrant workers and aimed to understand the experiences and perceptions of migrant workers and their lives in Singapore. The research questions were: (a) What are the sources of stress encountered by migrant workers? (b) How does the socio-structural environment create stress for migrant workers? (c) How do migrant workers deal with stresses encountered in their everyday lives?

### Participant sampling and recruitment

Migrant workers were eligible for the study if they were (i) aged at least 21 years; (ii) held a Work Permit pass or Special Pass card at the time of recruitment; (iii) not employed in household domestic labour; and (iv) consented to being audio-recorded. Special Pass cards are issued to migrant workers who no longer have working rights in Singapore but remain in Singapore for specific purposes such as due to an injury or salary claim from their employers. Those who did not meet these inclusion criteria were excluded. Initial contact was facilitated through a national non-governmental organization (NGO) providing subsidized medical, dental and mental health services for the migrant worker community. We obtained permission from the NGO to distribute invitation letters in English and major native languages (i.e., Bengali, Chinese, Tamil) to migrant workers attending clinics.

We undertook an initial passive recruitment strategy with no direct approach of workers, to minimize undue influence. Migrant workers were recruited into the study when they contacted the study team to indicate their interest to participate in the study. We then used snowball sampling by asking participants to inform their friends and colleagues who might be interested in the study and refer those interested to the study team. We used this approach to enable recruitment of migrant workers who were not users of the NGO’s medical services, as those attending the clinic typically were in poorer health and required medical services. Recruitment was carried out concurrently with data analysis and ceased when our analysis indicated data saturation had been achieved [52, 53].

### Consent process

Before each interview, we explained study objectives to participants, informed them that participation would not affect their employment or services received and obtained their written informed consent. All participants were given a copy of the study information sheet and consent form.

### Data collection

We developed a topic guide to steer semi-structured interviews (SSIs) while allowing deeper exploration of issues that arose. Existing literature on the lived experiences of migrant workers was used to guide development of its conceptual domains [47–49, 54], encompassing: (a) individual life experiences (events that elicited stress); (b) community experiences (individual and collective social capital); (c) health (mental and physical health concerns); and (d) stress response behaviours.

Individual and group interviews were conducted by trained researchers (i.e. HY, AF, AL). Group interviews were conducted due to difficulties in scheduling individual interviews with some participants and to accommodate their limited availability. All interviews were conducted in private spaces at the NGO office or quiet, mutually agreed settings (e.g., cafes) where privacy could be ensured. All interviews were conducted in English [30] or in Tamil [4] by AF. No other interview languages were requested by participants. Each individual SSI lasted approximately 60 to 90 min while group interviews lasted between 90 to 120 min. All interviews were audio recorded, and recordings transcribed verbatim, or translated and transcribed, by a professional transcriptionist. AF checked all transcripts against audio recordings for accuracy. Each participant was given supermarket vouchers worth SGD 10 as a gesture of appreciation in accordance with National University of Singapore Institutional Review Board guidelines.

### Data management

All participants were assigned a unique study identifier to preserve confidentiality. No personal identifiers were collected and any noticeable personal identifiers mentioned (e.g. names, locations) were permanently redacted during transcription. All data were stored in a secure encrypted computer, only accessible to the research team, and archived or disposed of in accordance with the National University of Singapore (NUS) data management policy.

### Data analysis

Transcripts from individual and group interviews were analyzed together. AF and AL independently conducted initial analysis using NVIVO 11 software (QSR International Pte Ltd), informed by Corbin and Strauss’ grounded theory [55]. They first familiarized themselves with transcript data then conducted open coding and line-by-line analysis of selected transcripts to identify data categories. These categories described participants’ interactions with structural conditions and explained how and why they handled stressful situations. Third, they conducted axial coding to identify and compare relationships between categories and organize them under higher order categories. Finally, they conducted selective coding to identify central categories that linked all categories together coherently [56]. Discrepancies were discussed and resolved collaboratively. Throughout analysis, researchers employed constant comparison with new data iteratively compared with existing data and existing categories to refine and create a representative set of categories. Categories were further refined and finalized through discussions between research team members to agree with final themes and interpretations. Data saturation was achieved when no new categories were generated from the data.

### Reflexivity

The researchers, three women and one man, were experienced community workers who have worked with marginalized communities. AF is a public health practitioner and PhD candidate who has worked extensively with domestic and non-domestic low-wage migrant workers in Singapore. AL is an anthropologist who has worked with migrants in Singapore and the United Kingdom. HY is an assistant professor with over ten years’ experience conducting community-led participatory research with marginalized communities in various countries. NH is an associate professor with over twenty years of applied public health research experience in the Asia region. All researchers are experienced in conducting research with vulnerable populations. Prior to the study, AF and AL underwent additional training on research ethics for vulnerable populations conducted by HY.

As data collectors, AF and AL undertook stringent measures to maintain data quality, documented in a “reflective diary,” memos and field notes following each interview to identify and mitigate any preconceptions. Prior to the study, both volunteered with the NGO as clinic assistants and interacted with migrant workers visiting the clinic. Cognizant of power relations and potential power imbalances in participant interactions, both worked together closely to compare notes, reflect on positionalities, and challenge viewpoints to ensure the rigor and trustworthiness of data collection and analysis were upheld.

### Ethics

The National University of Singapore Institutional Review Board provided ethics approval (Reference NUS-IRB-S-19–190).

## Results

### Interviewee characteristics and schematic map

Table 1 provides characteristics of the 34 study participants, 21 from individual and 13 from two group interviews with 8 and 5 participants respectively. There was no overlap of participants between group and individual interviews. All participants were male, 74% were aged 20–30 years old and 26% were aged 30–40 years old. About 82% of participants were Bangladeshi, of whom, 86% lived in dormitories and the remaining 14% lived in private accommodation. The remaining 18% of the participants were Indian, of whom, 67% lived in dormitories and 33% lived in private accommodation. About 91% of participants worked in the construction sector and 9% worked in the marine shipyard sector.

**Table 1:** Participant characteristics

ID	Gender	Home Country	Job Sector	Residence	Language of Interview	Approximate Age
INDI_P01	M	Bangladesh	Construction	Dormitory	English	20 – 30
INDI_P02	M	Bangladesh	Construction	Dormitory	English	20 – 30
INDI_P03	M	Bangladesh	Construction	Dormitory	English	20 – 30
INDI_P04	M	Bangladesh	Construction	Dormitory	English	20 – 30
INDI_P05	M	Bangladesh	Construction	Dormitory	English	20 – 30
INDI_P06	M	Bangladesh	Construction	Dormitory	English	20 – 30
INDI_P07	M	Bangladesh	Construction	Dormitory	English	20 – 30
INDI_P08	M	Bangladesh	Construction	Dormitory	English	20 – 30
INDI_P09	M	Bangladesh	Construction	Dormitory	English	30 – 40
INDI_P10	M	Bangladesh	Construction	Dormitory	English	30 – 40
INDI_P11	M	Bangladesh	Construction	Dormitory	English	30 – 40
INDI_P12	M	Bangladesh	Construction	Private	English	30 – 40
INDI_P13	M	Bangladesh	Marine & Shipyard	Dormitory	English	30 – 40
INDI_P14	M	Bangladesh	Marine & Shipyard	Dormitory	English	20 – 30
INDI_P15	M	India	Construction	Dormitory	Tamil	30 – 40
INDI_P16	M	India	Construction	Dormitory	Tamil	20 – 30
INDI_P17	M	Bangladesh	Construction	Dormitory	English	20 – 30
INDI_P18	M	Bangladesh	Construction	Dormitory	English	30 – 40
INDI_P19	M	India	Construction	Dormitory	Tamil	30 – 40
INDI_P20	M	India	Construction	Dormitory	Tamil	20 – 30
INDI_P21	M	Bangladesh	Marine & Shipyard	Dormitory	English	20 – 30
GI1_P22	M	Bangladesh	Construction	Private	English	20 – 30
GI1_P23	M	Bangladesh	Construction	Dormitory	English	20 – 30
GI1_P24	M	Bangladesh	Construction	Dormitory	English	20 – 30
GI1_P25	M	Bangladesh	Construction	Dormitory	English	20 – 30
GI1_P26	M	Bangladesh	Construction	Dormitory	English	20 – 30
GI1_P27	M	Bangladesh	Construction	Dormitory	English	20 – 30
GI1_P28	M	Bangladesh	Construction	Dormitory	English	20 – 30
GI1_P29	M	Bangladesh	Construction	Dormitory	English	20 – 30
GI2_P30	M	Bangladesh	Construction	Private	English	30 – 40
GI2_P31	M	Bangladesh	Construction	Dormitory	English	20 – 30
GI2_P32	M	Bangladesh	Construction	Private	English	20 – 30
GI2_P33	M	India	Construction	Private	English	20 – 30
GI2_P34	M	India	Construction	Private	English	20 – 30

*INDI* Individual Interview, *GI* Group Interview, *PXX* Participant Number, *E.G. GI1_P22* Group Interview 1 Participant Number 22

**Fig. 1:**
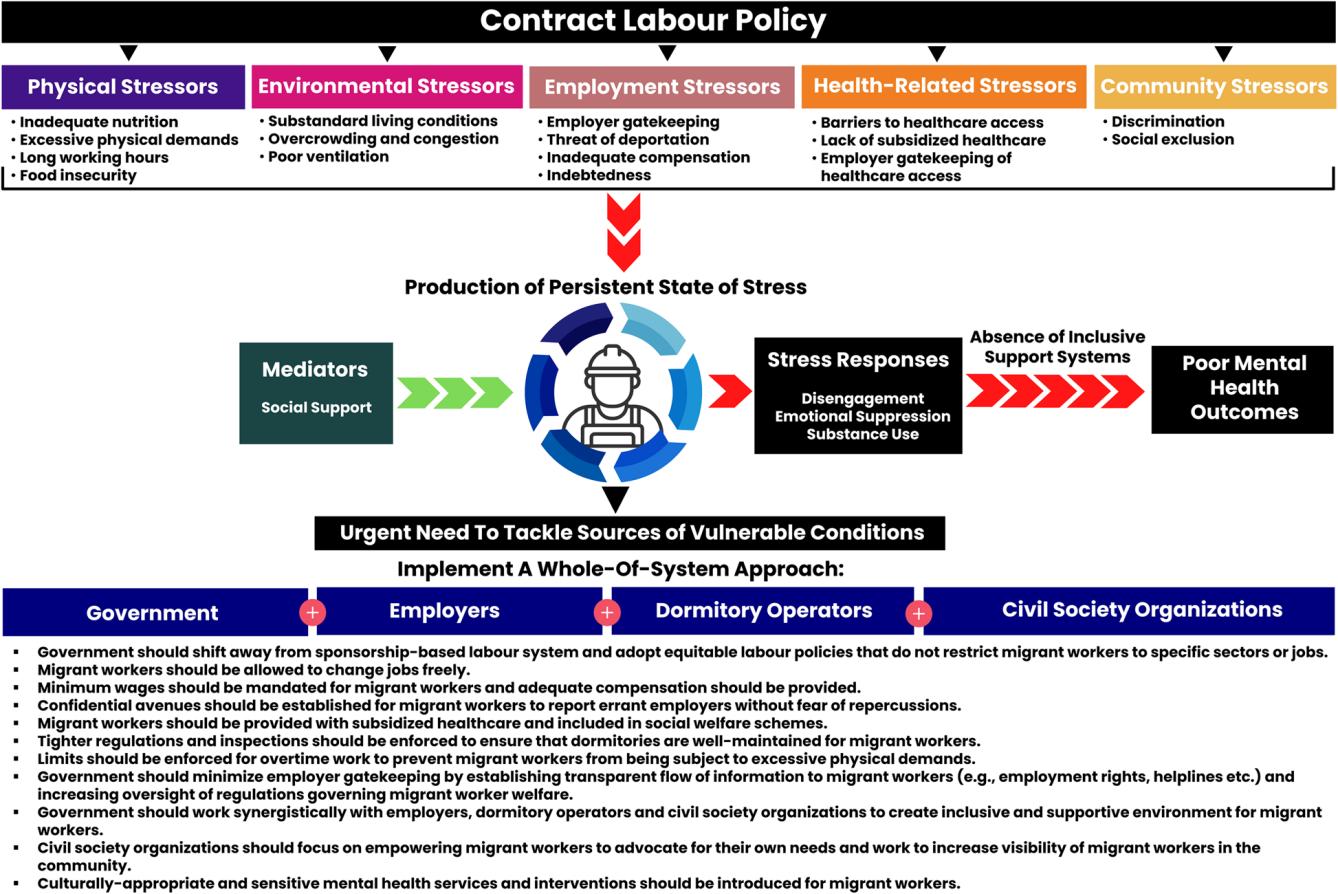
Sources of social vulnerabilities, resulting stressors and effects on low-wage male migrant workers in Singapore

Figure 1 shows the schematic map of how we constructed vulnerability in this migrant worker population and sources of these vulnerabilities. We found the conditions of vulnerability experienced by migrant workers were predominantly a product of structural factors within the migrant labour system. Migrant workers experienced a multitude of stressors created by socio-structural factors, which acted synergistically with stressors arising from their environmental circumstances to create conditions of vulnerability. Migrant workers exercised agency in attempting to tackling these stressors but were embedded in a cycle of chronic stress driven by their social environment as low-wage foreigners in Singapore.

Our analysis identified three categories of: (i) multiple stressors (sub-categorized as environmental, physical, employment, health-related, and community); (ii) mental health effects of constructed vulnerability; and (iii) migrant worker mitigatory responses.

### Multiple stressors

#### Environmental

Most participants reported substandard living conditions as a key cause of vulnerability. In Singapore, housing for migrant workers—such as purpose-built dormitories—is overseen by the Building and Construction Authority (BCA) that mandates minimum living standards for migrant worker accommodations [57]. Though the BCA requires at least 3.5 sqm of living space excluding common areas to be allocated per migrant worker, this is difficult to enforce in dormitories, where profit motivations may cause dormitory managers to lease out bedspaces to as many workers as possible. Participants described being housed in congested and poorly ventilated living spaces shared with 12 other men on average. Many described their rooms as having insufficient ceiling fans or windows to allow proper ventilation. This created humid and uncomfortable conditions that negatively affected their sleep, with many suffering from insomnia and chronic fatigue as they were unable to get adequate rest. Participants also described having inadequate personal storage areas, causing them to store their belongings around their beds, resulting in cluttered living spaces.

*“[The] dormitory [does] not allow us to bring own fan or something like that, like 12 person have one ceiling fan […]. So, you know it’s too hot. [We are] migrant worker[s] right, so some people are doing lots of hard work under the sun. It’s too hard. Once we come back to home..[…] and want to sleep, we can’t sleep. It’s too hot […] we [are] all sweating. Like our bed, everything, it’s too sweaty. End of the day we become sick already. Even the environment of the room is [not suitable]. […] But once you [have] 12 persons, 15 persons… 2m-by-2m-by 2m, 3m-by-3m-by-3m square room like […] it’s not really good. Because […], once the more people together, the environment also changes. It’s like the lack of oxygen. Some time, you could feel the lack of oxygen. You know like the humidity, like the [heat].”* – INDI_P05

#### Physical

Poor nutrition and excessive physical demands further perpetuated chronic stress and fatigue. While some dormitories had cooking facilities, many did not provide spaces for migrant workers to cook their own meals. Most participants lived in dormitories that did not allow cooking and had to rely on catering companies for their meals. However, catered meals were described as nutritionally inadequate. As a result, participants reported consuming energy drinks to get sufficient energy for work. Many also described losing significant weight due to poor nutritional intake.

*“This food [is] totally unhealthy, no [taste] also. It’s only for [staying alive], not dining. Just eating for life, to stay alive. No energy. If you [eat] the unhealthy food, mental planning also cannot [do]. No energy, I cannot [think about] anything. […] When I first [came here], my weight is 65 kg. But when I [went] back, my weight is 40 kg. It’s a lot of hard work in there. […] This catering food cannot eat all. Little bit only can eat*. *[That’s why we drink] Red Bull, [lots of] energy drinks. That’s why when [we] go back Bangladesh […] If you stay [in Singapore for] 14 years, after 20 years we’re [getting] a lot of disease. Cancers, kidney disease.” –* INDI_P06

Reported substandard food preparation and hygiene practices of catering companies and a lack of proper storage areas for catered food at worksites led to frequent experiences of food spoilage and chronic food insecurity.

*“Sometimes […] the curry already smell. Then I throw. Then I [just] eat rice. […] These [caterers] think, 12 hours people can still eat. But in 12 hours […] food can spoil. […] And this food when we take to the workplace, then they provide one box only for us to keep on site. […] Then when we open this food at 12 o’clock, spoilt, bad smell, cannot eat. Then after that many people throw. […] sometimes spoilt also you makan [eat], because [you have] no choice, [we] cannot buy also […] no time, maybe [we] don’t have money. I saw some people [do this] but they suffer. [Most of the time] curry spoil, because some vegetables cannot be in good condition after 3-4 hours not eaten*.” – INDI_P07

Participants were subjected to long working hours and heavy physical demands at worksites despite chronic food insecurity and deficient nutritional intake. As a result, many described chronic pain and health issues, contributing to negative appraisals of their quality of life.

*“Actually, like, we’re working 12 hours or 13 hours, 14, 16 hours… I’ve experienced [working] […]30 hours sometimes. I have no sleep, no food. But you know we’re long-term working. Sometimes we feel back pain, sometime migraine […] too much headache. Too much pain. Sometimes we can feel it. Sometimes we can see our own self. My hair is going to falling. Lots of the symptoms, we can see nowadays. Then end of the time, I don’t know what’s happened. Some time you know like, very hard to breathing. Very hard to breathe in sometime.”* – INDI_P05

#### Employment

Migrant workers’ right to live and work in Singapore was tied to employer sponsorship, which put them at heightened susceptibility to exploitation. This absolute control wielded by employers as “gatekeepers” resulted in power imbalances, which disempowered migrant workers from voicing any displeasure in public about their employers. Participants expressed fear of speaking against their employers in case they were deported to their countries if they complained.

*“Because number one problem we are very scared to complain […], anyway because we work here, we take care of our family. Our family depends to me…[…] [I’m] scared the company catch me, send [me back] to Bangladesh and India […] So this thing also we cannot go complain. Because [if] I go complain, company make rules.”* – INDI_P13

Power imbalance enabled some employers to adopt coercive practices. Participants reported abuse of power and pressure from employers to work long hours and coercive practices such as docking pay to discourage workers from accessing essential needs such as medical care.

*“My first company same, if sick right, fever also have to meet [at] the company office, the boss, after the supervisor sit down. They don’t care. You die also don’t care like sick right, fever right, [you have to wait]. When finish his job, then after slowly bring [you to see doctor] […]. [Even if we go] to see company doctor also, he cut money. Many more 50 dollar 60 dollar that [employer] cut, so we come to here just time lost only. […] We go company doctor […], but when my salary come in then they cut.”* – INDI_P15

Participants were unwilling to speak openly against such coercive practices as the threat of deportation was a constant worry. They feared losing their jobs due to financial commitments back home and high indebtedness. Almost all participants had borrowed money or obtained bank loans by mortgaging their assets in their home countries to pay recruitment agents for jobs in Singapore. Thus, they faced constant pressure to pay back loans and provide financially for their families.

*“When we’re coming to Singapore, first problem is the agent fee. We have to pay a lot of agent money. More than $10,000. After one year, the company permit, the contract is one year. So, after one year, company will send [us back]. Maximum two, three [years] [if] company [allows] only [that we] can stay. But [most of the time] company can send back. So, we go back again. Again, second time, we need to come [to Singapore]. Then again, need to pay the same. $8000 like that. […] So, [need to] take the bank loan. [Every] month need to pay; bank need to pay.[…] some company don’t give the salary timely.* […] *So, in this time, we don’t have money to top up. We have a lot of loan in our country.[…] So we have to pay, every month have to pay. Eat or don’t eat also doesn’t matter.”* – INDI_P06

Inadequate compensation for their work added to the pressure participants faced, resulting in them taking overtime work to meet financial obligations. This led to a vicious cycle of migrant workers working long working hours with minimal pay to fulfil their financial commitments.

*"And why I continue working 9 years in Singapore – this one meaning is what? Money not enough my family. Why? They give me basic salary $18 [per day]. One hour I working give me $2. […] one hour I working the hard job, just give me $2. Actually, very bad […] I buy Singapore everything [more expensive now]. So… [but] my salary is same, $18, why?* […] *I know I worker. So workers is like [also] have life. I have life, I use the phone. I have family, I makan [eat]. Today, I buy this t-shirt. […]. So $40 [for the shirt], my – how many days salary? How many days?* […] *This one only buy, I two days salary gone already. […] Together, 9 years I working, maybe my balance five thousand also not enough. Nine years meaning what? My young life, finish[ed] already?”* – GI1_P27

#### Health

Another cause of heightened vulnerability for participants was facing barriers to accessing healthcare to preserve their health and employability. While employers were required to purchase mandatory medical insurance for hospitalizations and work injury compensation, this did not extend to workers’ general medical needs such as primary and preventive care. As non-residents, migrant workers were excluded from subsidized healthcare in Singapore, resulting in high out-of-pocket costs for acute medical treatment. Employer gatekeeping is also common, with migrant workers having to obtain approval from supervisors prior to seeking medical care. Though employers were responsible for the costs of providing medical treatment for migrant workers, regardless of whether conditions were work-related, having to obtain employer approval to seek medical care presented an enduring barrier to seeking care for many migrant workers. Most participants recounted difficulties obtaining approval from their employers to seek medical treatment or get reimbursed for medical costs.

*“Polyclinic very expensive. When I went the first time, $200. First time company gave the $200. Second time it was $160, company only gave the medicine money, not the check-up money. Half give. […] when I got injured that time, I first got treatment from company clinic. Then no improvement, no recover from my injury. Then this company transferred me to the Changi General Hospital, so this needed the LOG [Letter of Guarantee] and need to pay money. So sometimes I go, boss didn’t give the LOG or money also, so I cannot take treatment.”* – INDI_P09

#### Community

In addition to chronic environmental stressors, migrant workers were subjected to stresses due to their non-resident status in Singapore. Migrant workers were considered transient and had no access to permanent residency or citizenship in Singapore. Work Permit passes have an expiry of 2 years unless renewed by employers, preventing their establishing roots in Singapore [30]. This created a sense of “uprootedness” among migrant workers as they lacked opportunities to assimilate into communities and forge a sense of belonging. Participants reported acute awareness of their foreigner status and consciousness of the negative sentiments associated with their identity, sharing anecdotes of being subject to social exclusion due to their “lower” status.

*“Because […] people […]don’t really like the migrants. They [think] like, “the migrant workers, they have to work. The migrant workers, why they are here?.” I have really [felt this before]…[get] lots of [comments]. Like last time […] I’m talking with some of people.[…] They’re commenting like, “oh migrant worker, why you are here? Why are you a part of [Singapore]? This, that. Is that really where he [belong]? Last time I have lots of experience like the locals don’t want to talk to us. Even they don’t want to sit beside, behind us [on the] MRT, bus they don’t want to sit with us. […] Because we don’t have much rights also. There is a lot of gaps of human rights also. But in Singapore, the environment of the migrants […] they don’t really care about migrants because we are not Singaporeans.”* – INDI_P07

Most participants had first-hand experience of discrimination linked to their non-resident status. Many of these discriminatory events were in social or work settings in which they interacted with the resident population.

*“[You] see the workers [on the] MRT or the bus. Anywhere. Because they finish work and they [are] going back […] home. [Sometimes], [their] body […] is smelly. Sometimes [they are] uncomfortable […] hungry. [The residents] look at us like…smelly, [disgusting]. [We] don’t say anything.”* – INDI_P04

Although many participants shared first-hand experiences of discrimination in social settings, possible encounters with government authorities and law enforcement officers were perceived as most stressful. Negative encounters of discrimination and perceived fear of wrongful persecution generated persistent fear and stress among participants.

*“Because [sometimes] we scared of the law also. Because the law says that if any of my friends fight together, then each of [them] will be transmitted [deported]. So, there’s no justification. Police just straightaway transmitted. There’s no reason, they can write anything. Because the law is made by them. They write anything, if they can [make] excuses and everything, just transmit [deport] and send back home.”* – INDI_P04

This led to hypervigilance and constant anxiety to “not get in the way” of the resident population. Participants expected to be “rejected” and treated as outsiders due to their minority characteristics. Expectations of deportation were an additional expectation of rejection experienced by migrant workers, as a result of restrictive labour laws. Additionally, participants noted being cognizant that the system served the interests of the majority and actively sought to minimize any conflict that would jeopardize their ability to continue working in Singapore.

*“Because like we don’t want arguments …[…] No one complaining about anything.*

*[…] Cannot, this one. Cannot fight, cannot shout also. Cannot disturb other people. Singapore law, everybody must take care everybody, everything.”* – GI1_P29

As a response to perceived discrimination and social exclusion by the Singaporean community, migrant workers often delayed seeking help or services to minimize contact with structural establishments. This led to delays in seeking care to avoid having to disclose any medical conditions to healthcare service providers and employers. In particular, due to hypervigilance and awareness of the repercussions of not ‘fitting’ into the system, participants often concealed conditions that could put them at risk of losing their jobs. While this minimized threats to their job security, the stress associated with poor health affected participants negatively.

*“[…] Before many of them, 7 or 8 [workers] got diabetes, then company said permit no renew, you go back.”* – INDI_P19

Apart from limiting healthcare access, concealment also hindered positive mental health responses as participants refrained from sharing the hardships they encountered with their families. This contributed to social isolation and limited support networks.

*“ […] We cannot talk also, [to] family because my mother, [my] father old already. If I talk, they feel [more] sick. Ah how my son. [Their]heart [might] stop (feel heartbroken), or they will [get] sick. [If we suffer from any] injury, any accident also we [most of the time] never talk to [our family about it]. They [will] keep [worrying]. If maybe full day no talk, night-time they will [ask] why today no talk. Maybe […] something happen [to me but] I say I busy, busy to work, but I never say my finger [got cut].”* – INDI_P14

Generally, migrant workers were stuck in a vicious cycle of persistent chronic stress, generated by enduring socio-structural factors. Persistent fatigue from working long hours, inadequate compensation and accommodation, poor nutrition, barriers to preserving their health and employability and constant fear of deportation were mutually reinforcing factors that acted synergistically to make migrant workers more vulnerable to adverse health outcomes.

### Effects of constructed vulnerabilities on mental health of migrant workers

As a result of the stressors encountered in their everyday lives, participants reported high levels of stress, which adversely impacted their mental health.

*“If I talk about my mental health, it’s like worse. Sometime like after I do the work […] I don’t want to talk. I don’t want to talk with my family. I don’t want to talk with my friends…[…] You know I cannot control myself sometimes. It’s stressful […].”* – INDI_P04

However, migrant workers lacked supportive environments to help them manage their stressors and avenues to seek help without the fear of repercussions. As a result, many participants felt isolated and lonely with few avenues to process their emotions.

*“I know the loneliness. When a person become more lonely, no have friends or anything, that time will [get] addicted to drugs, smoking, alcohol. This time will destroy their lives. […] So, this time they’re feeling very… tension. […] just coming work, then go room sleep. Work, sleep. Lonely, boring. They have no friends. Very lonely, nobody come and [talk] together. Very lonely. Some people suicide also. Mm… a lot of people during midnight sleep go out [of their room] crying. Loudly crying [because they miss family]. No way to go [back]. That’s why feeling very lonely. Understand some people [commit] suicide [because of] loneliness.”* – INDI_P06

## Responses and coping mechanisms

As the stressors encountered by migrant workers were enduring fixtures of the social structure in which they were embedded, we attempted to identify how participants responded to regulate negative effects. We found that social support was a key mediator in alleviating stress. Participants shared that having friends and support networks in Singapore helped mitigate stressors and provide some respite. Participants also sought connections on social media, such as Facebook, to connect with their countrymen and make friends. Social networks also helped participants provide support for one another and navigate the stressors they encountered in Singapore.

*“[…] we [try to] make friends. Sometime here […]all musical […] musical [events]. Then we see sometime the Facebook, we see all our Bangla people, doing the song [taking part in musical events]. Then we come [together]…join us […]. Like that, like this then make already the friend.[Sometime], I going outside, somebody say, “hi brother, how are you?”* – GI1_P25

Most participants tried to manage chronic stress by actively diverting their attention to other matters and distracting themselves from thinking about their stressors.

*“We make ourselves busy with the activities, so that we don’t really feel that we’re alone here. Or other issues.”* – INDI_P01

Participants also kept their feelings associated with the stressors they encountered to themselves and rarely vocalized their feelings. Some shared that they did not want to worry their friends and families by communicating their difficulties to them.

*“Because I keep inside everything, because I cannot call [and tell] my family also. [If] I call [my] family everybody [will be] thinking about me, cry, they are sad. I also cannot go [back to home country] because I am working here.. I take care of my mother, my father, everybody…we cannot talk to [anyone]……Just we keep [everything] in the heart inside. Because our pain we are the [only ones] who understand…We cannot complain anywhere.”* – INDI_P13

A few participants also engaged in harmful stress responses such as smoking and drinking alcohol as an outlet to release accumulated stress.

*“Then […] he have ten thousand loan [to come to] Singapore. He take from somebody loan. He cannot give [back]. He had a lot of drinks, smoking. A lot of things. […] Some people [have] family crisis. Family crisis and the loneliness. […] [keep] drinking more, drinking*.” – INDI_P06

Participants predominantly engaged in stress responses that focused on moderating the emotions triggered by stressors instead of actively alleviating their stressors, as they were aware that most stressors could not readily be changed.

## Discussion

This qualitative study critically examined the sources of vulnerability that predispose low-wage migrant workers to increased risks of poor health outcomes in Singapore, a major labour-receiving country using a social stress perspective. Through the mapping of the underlying sources of vulnerability and ensuing stressors faced by migrant workers, we identified a persistent cycle of precarious employment, material and social deprivation and a lack of social protection networks for migrant workers. This is aligned with similar studies that found migrant workers facing social, cultural and structural barriers in accessing essential services, underscoring the need to tackle gaps in health and social protection for migrant workers [58–60].

We found that Singapore’s contract migration model was the primary cause of the various vulnerabilities that migrant workers encountered in Singapore. Singapore adopts a bifurcated labour migration model where unskilled and low-skilled migrant workers are treated as a transient workforce subject to restrictive measures that bar them from long-term integration into the community [61]. This includes imposing a sponsorship system that ties these workers to employer-sponsored Work Permit passes with 2-year validity periods, that effectively limit migrant workers from changing employers or job sector without their sponsor’s approval [41]. Further, this sponsorship system endows employers with gatekeeping power over migrant workers’ lives and the ability to restrict workers’ economic mobility and autonomy. This power imbalance propagated by Singapore’s labour regime leaves migrant workers vulnerable to economic exploitation. In receiving countries that adopt restrictive sponsorship-based labour practices like Singapore, such as the Gulf Cooperation countries, mistreatment and abuse of workers have been widely reported [62–65]. This signals the need to shift away from the restrictive sponsorship system and employer gatekeeping to give migrant workers the right to change employers freely if they experience issues. While reforms to the sponsorship system have been introduced in some Gulf countries, such as removing the requirement for employers to consent to workers’ job changes [66], more efforts are needed to improve labour protection for migrant workers in Singapore.

The sponsorship system further created job precarity as the short-term Work Permit passes allowed employers to deport migrant workers easily without consequence. Threats of deportation and losing their jobs were constant sources of worry for migrant workers who were sole breadwinners for families in their home countries and negatively impacted their mental health. This was in alignment with existing studies from Singapore which looked at the mental health of South Asian migrant workers in Singapore [67, 68]. For instance, in a 2015 study by Harrigan et al., 10% of sampled regular Work Permit holders and 64% of sampled workers who were awaiting compensation for injury and salary claims stated that they had experienced threats of deportation from their employers. From this group, 48% of regular workers and 70% of salary and injury claim workers reported high scores on the Kessler-6 scale, indicative of serious mental illness [47]. These findings warrant the need to tackle labour malpractices and minimize employer gatekeeping of workers. Furthermore, it is crucial to look into the mental health needs of migrant workers and provide safe avenues for migrant workers to seek assistance.

Aside from the precarious nature of their employment, migrant workers also experienced chronic physical stressors. Overcrowded living conditions and impacted sleep quality contributed to chronic fatigue. Poor sleep quality has been associated with higher risks of workplace injuries, in particular, falls from heights, fractures and dislocations among migrant workers [69–72]. For instance, in Singapore, a total of 11 fatal falls among migrant workers were reported in 2020 with the construction industry accounting for most fatalities despite safety measures, raising alarm over the workplace safety of migrant workers [73]. Poor sleep quality has been linked to depressive symptoms and psychological distress among migrant workers as well [70, 72, 74]. In addition to affecting sleep quality, overcrowded living environments contributed to the rapid spread of COVID-19 in purpose-built dormitories in Singapore, resulting in migrant workers accounting for more than 90% of the total COVID-19 caseload in 2021 [75]. These issues underscore the urgent need to improve living spaces for migrant workers and enforce adequate regulations for the maintenance and upkeep of migrant worker accommodations.

Consistent with findings from other receiving countries, exclusion from universal health coverage and barriers to healthcare contributed to vulnerabilities encountered by migrant workers in this study [9, 18, 19]. Migrant workers’ exclusion from subsidized healthcare and financial assistance schemes in Singapore puts them at risk of high out-of-pocket medical costs and financial hardship [38, 39, 49]. In addition, migrant workers are also restricted from seeking medical care freely due to employer gatekeeping of healthcare, contributing to delayed health-seeking [76]. Delayed health-seeking behaviour among migrant workers has been associated with increased chronic disease burden and reduced quality of life [77–79]. Hence, it is important to push for inclusion of migrant workers in universal health coverage schemes in receiving countries and tackle barriers to healthcare access.

Apart from the compounding vulnerabilities related to socio-environmental circumstances, our findings also illustrated that migrant workers already disadvantaged by structural exclusions faced additional stress due to their non-resident status. Migrant workers in our study reported experiencing discrimination and social exclusion due to their identity as “foreigners” and prevailing “anti-foreigner” sentiments in the community. This “othering” of labour migrants has been observed elsewhere [80, 81]. In Gulf countries, abuse and discrimination against migrant workers are common, with workers subjected to social exclusion, neglect and denial of essential services [82, 83]. This emphasizes the need to tackle discrimination against and othering of migrant workers and create an inclusive social environment. Moreover, migrant workers were found to internalize their identity as “foreigners,” which further propagated negative perceptions that they were not meant to assimilate despite their contributions to the Singapore economy [30, 61]. Internalization of discriminatory events and negative stereotypes have been closely linked to individual psychological distress [84], emphasizing the need for policy interventions to improve social inclusion for migrant workers in receiving countries.

### Stress responses and mental health outcomes

Migrant workers in our study resorted to a variety of stress responses to mitigate the effects of stress. Social support was cited by participants as a protective strategy to help alleviate stress. Having adequate social support is protective against poor mental health in migrant worker communities [85, 86]. Studies have found that negative effects of migrant workers’ experiences of discrimination and stress were attenuated by inclusive social support systems [85–89], underscoring the importance of strengthening the social capital of migrant workers to build their resilience against stressors.

We found that migrant workers predominantly focused on self-management of their emotions and felt disempowered to actively tackle sources of stressors. This was likely due to workers being aware that many stressors arose from structural factors beyond their locus of control. As a result, migrant workers learnt to negotiate the social system in which they were embedded and exercise control over their internal emotional state instead. Focusing on managing one’s emotions in circumstances that are unchangeable has been found to be protective against psychological distress in the short term, but prolonged use of such emotion-focused coping strategies to mitigate effects of persistent stress can exacerbate negative self-appraisal long term [90–92]. Thus, it is imperative to provide avenues for migrant workers to seek social support and resources to help manage stressors.

It is worth noting that, although migrant workers are governed by a labour system that perpetuates the cycle of hardships they face, they should not be seen as powerless or lacking self-efficacy. Migrant workers in our study exercised self-efficacy to manage hardships and cope with stressors. They endeavoured to navigate structural stressors and focus on their purpose for seeking employment in Singapore – earning money to support their families at home. Providing a better life for families back home was the primary motivator that pushed migrant workers to manage hardships encountered in Singapore [54, 93, 94]. Thus, while we recognize the political and economic interests of receiving countries in restricting the import of foreign labour to maximize benefits, there is much to be gained by adopting more rights-centered approaches to avoid unintended exploitation [95, 96]. The COVID-19 pandemic underscored the importance of protecting vulnerable communities and investing in making health systems resilient and inclusive to all, including addressing gaps in health and social protection for migrant workers.

Since the COVID-19 pandemic, significant policy reforms have been introduced in Singapore to address some of the issues highlighted. For instance, a primary care health system coupled with a primary care insurance plan for all migrant workers was introduced in November 2021 [97, 98]. Under this system, migrant workers will be enrolled with a medical service provider in regional medical centres depending on their location of residence and are able to access medical care more easily. In addition, employers are required to purchase primary care insurance plans that covers the cost of medical consultations and treatments at these medical centres, with migrant workers co-paying only SGD5 dollars for consultations. These policy reforms help minimize barriers to health-seeking for migrant workers. Furthermore, the Singapore government expanded the Foreign Employee Dormitories Act (FEDA), which imposes minimal living standards for migrant worker accommodations to all dormitories regardless of size in September 2022 [99, 100]. Prior to this reform, the FEDA only covered dormitories that housed at least 1000 workers. This reform would allow for stricter regulation of all dormitories under a single law and improve living standards for migrant workers. These policy reforms are commendable and timely, though it remains important to also tackle the sources of precarity that create and reproduce social vulnerabilities among migrant workers. More needs to be done to dismantle exploitative labour policy systems globally and tackle unequitable policies that lead to disparate health outcomes for migrant workers. It is crucial to sustain the momentum for change and work towards building more resilient and inclusive health systems for all.

### Limitations

Study limitations should be considered in interpreting our findings. The views expressed by the participants may not fully represent the lived experiences of all migrant workers in Singapore as recruitment was mainly confined to NGO clinics and key informants from the migrant worker community. In addition, the cultural differences in managing stress were also not explored in this study. The participants recruited for this study were mainly from India and Bangladesh and did not cover workers from other labour-exporting countries like China, Myanmar and Thailand who make up a substantial proportion of the low-wage foreign labour workforce. However, migrant workers from India and Bangladesh constitute the majority of the pool of migrant workers in Singapore. Nonetheless, further research is needed to expand on the sample population and to elucidate the construction of vulnerabilities and cultural influences on coping strategies of migrant workers from different countries of origin. Nevertheless, our study contributes to a limited body of evidence on the linkages between structural sources of stress and vulnerability among low-wage migrant workers in Singapore.

## Conclusions

Our findings illustrate how social vulnerabilities are constructed among migrant workers and the interaction of various sources of stress. We found that conditions of stress experienced by migrant workers were predominantly a product of mutually reinforcing socio-structural and environmental factors, creating chronic stress. Our findings highlight the need to address the mental health burden placed on workers by continuing to improve labour and social protection for the migrant worker population. It is crucial to create avenues for migrant workers to seek social support and engage stakeholders, such as employer and civil society actors, to improve access to support services. It remains crucial for stakeholders to collaborate both nationally and globally on health equity and build collective resilience among migrant workers.

## Supplementary Information

**Additional file 1.** Semi-Structured Interview Guide.

## Data Availability

The datasets generated and analysed for this study are not publicly available due to the risk of identifying participants but are available from the corresponding author upon reasonable request.

## Abbreviations

BCA: Building and Construction Authority
COVID-19: Coronavirus Disease 2019
EP: Employment Pass
FEDA: Foreign Employee Dormitories Act
GI: Group Interview
INDI: Individual Interview
LOG: Letter of Guarantee
NGO: Non-Governmental Organization
NUS: National University of Singapore
SGD: Singapore Dollars
SSI: Semi-Structured Interview
WP: Work Permit

## References

[1] United Nations. International Migration 2020 Highlights. United Nations. Available from: https://www.un.org/en/desa/international-migration-2020-highlights. [cited 17 Jan 2022].

[2] Popova N. ILO Global estimates on international migrant workers: results and methodology. 2nd ed. Geneva: International Labour Organization; 2018.

[3] Ngan LLS, Chan KW (2013). An outsider is always an outsider: migration, social policy and social exclusion in East Asia. J Comp Asian Dev.

[4] White JA, Rispel LC (2021). Policy exclusion or confusion? Perspectives on universal health coverage for migrants and refugees in South Africa. Health Policy Plan.

[5] Moyce SC, Schenker M (2018). Migrant workers and their occupational health and safety. Annu Rev Public Health.

[6] Mucci N, Traversini V, Giorgi G, Garzaro G, Fiz-Perez J, Campagna M (2019). Migrant workers and physical health: an umbrella review. Sustainability.

[7] Mucci N, Traversini V, Giorgi G, Tommasi E, De Sio S, Arcangeli G (2020). Migrant workers and psychological health: a systematic review. Sustainability.

[8] Hasan SI, Yee A, Rinaldi A, Azham AA, Hairi FM, Nordin ASA (2021). Prevalence of common mental health issues among migrant workers: a systematic review and meta-analysis. PLoS ONE.

[9] Pham KTH, Nguyen LH, Vuong QH, Ho MT, Vuong TT, Nguyen HKT (2019). Health inequality between migrant and non-migrant workers in an industrial zone of Vietnam. Int J Environ Res Public Health.

[10] Devkota HR, Bhandari B, Adhikary P (2021). Perceived mental health, wellbeing and associated factors among Nepali male migrant and non-migrant workers: a qualitative study. J Migr Health.

[11] Fakhoury J, Burton-Jeangros C, Consoli L, Duvoisin A, Courvoisier D, Jackson Y (2021). Mental health of undocumented migrants and migrants undergoing regularization in Switzerland: a cross-sectional study. BMC Psychiatry.

[12] Mathew G, Ramesh N, Shanbhag D, Goud R, Subramanian S, Lobo C (2016). Quality of life and probable psychological distress among male workers at a construction site, Kolar district, Karnataka, India. Indian J Occup Environ Med.

[13] Lam KKF, Johnston JM (2015). Depression and health-seeking behaviour among migrant workers in Shenzhen. Int J Soc Psychiatry.

[14] Preibisch K, Hennebry J (2011). Temporary migration, chronic effects: the health of international migrant workers in Canada. CMAJ.

[15] Suphanchaimat R, Pudpong N, Tangcharoensathien V (2017). Extreme exploitation in Southeast Asia waters: challenges in progressing towards universal health coverage for migrant workers. PLoS Med.

[16] Guinto RLLR, Curran UZ, Suphanchaimat R, Pocock NS (2015). Universal health coverage in ‘One ASEAN’: are migrants included?. Glob Health Action.

[17] Satinsky E, Fuhr DC, Woodward A, Sondorp E, Roberts B (2019). Mental health care utilisation and access among refugees and asylum seekers in Europe: a systematic review. Health Policy.

[18] Liang Y, Guo M (2015). Utilization of health services and health-related quality of life research of rural-to-urban migrants in China: a cross-sectional analysis. Soc Indic Res.

[19] Uddin MS, Akhtar R, Masud MM, Hye QMA (2020). Utilization of health care services among migrant workers in Malaysia. Human Soc Sci Lett.

[20] Dressler WW, Oths KS, Gravlee CC (2005). Race and ethnicity in public health research: models to explain health disparities. Annu Rev Anthropol.

[21] Aneshensel CS, Rutter CM, Lachenbruch PA (1991). Social structure, stress, and mental health: competing conceptual and analytic models. Am Sociol Rev.

[22] Aneshensel CS (1992). Social stress: theory and research. Ann Rev Sociol.

[23] Meyer IH, Schwartz S, Frost DM (2008). Social patterning of stress and coping: does disadvantaged social statuses confer more stress and fewer coping resources?. Soc Sci Med.

[24] Pearlin LI (1989). The sociological study of stress. J Health Soc Behav.

[25] Meyer IH (2003). Prejudice, social stress, and mental health in lesbian, gay, and bisexual populations: conceptual issues and research evidence. Psychol Bull.

[26] Wheaton B (1985). Models for the stress-buffering functions of coping resources. J Health Soc Behav.

[27] Kaur A (2010). Labour migration trends and policy challenges in Southeast Asia. Policy and Society.

[28] International Labour Organization (ILO). Measuring labour migration in ASEAN: Analysis from the ILO’s International Labour Migration Statistics (ILMS) Database. 2022. Available from: http://www.ilo.org/asia/publications/WCMS_839321/lang--en/index.htm. [cited 29 May 2023].

[29] Ministry of Manpower Singapore. Foreign Workforce Numbers. Available from: https://www.mom.gov.sg/documents-and-publications/foreign-workforce-numbers. [cited 19 Jun 2023].

[30] Chin C (2019). Precarious work and its complicit network: migrant labour in Singapore. J Contemp Asia.

[31] Ministry of Manpower Singapore. Key Facts on Employment Pass. Available from: https://www.mom.gov.sg/passes-and-permits/employment-pass/key-facts. [cited 24 Jun 2023].

[32] Yeoh BSA, Baey G, Platt M, Wee K. Migrant workers and the politics of (im)mobility. In: Migrant Workers in Singapore. World Scientific; 2022 p. 3–5. Available from: https://www.worldscientific.com/doi/, 10.1142/9789811255038_0002. [cited 29 May 2023].

[33] Yeoh BSA (2006). Bifurcated labour: the unequal incorporation of transmigrants in Singapore. Tijdschrift voor Econ Soc Geograf.

[34] Council on Foreign Relations. What Is the Kafala System? Available from: https://www.cfr.org/backgrounder/what-kafala-system. [cited 29 Dec 2020].

[35] Transit Workers Count Too (TWC2). TWC2 survey: starting salaries for migrant workers flatlined for the last 10 years. Available from: https://twc2.org.sg/2017/01/15/twc2-survey-starting-salaries-for-migrant-workers-flatlined-for-the-last-10-years/. [cited 24 Jun 2023].

[36] Han K. Singapore’s Migrant Workers Struggle to Get Paid | CNN. Available from: https://edition.cnn.com/2018/02/24/asia/singapore-migrant-workers-intl/index.html. [cited 24 Jun 2023].

[37] Yeoh BSA, Wee K, Lam T. Migrant workers in Singapore: lives and labour in a transient migration regime. World Scientific; 2022. Available from: https://www.worldscientific.com/worldscibooks/10.1142/12798. [cited 29 May 2023].

[38] Rajaraman N, Yip TW, Kuan BYH, Lim JFY. Exclusion of migrant workers from national UHC systems—perspectives from HealthServe, a non-profit organisation in Singapore. Asian Bioethics Review. 2020. Available from: https://link.springer.com/epdf/, 10.1007/s41649-020-00138-y. [cited 12 Aug 2020]. 10.1007/s41649-020-00138-y PMC7396727 32837561

[39] Ang J, Koh C, Chua B, Narayanaswamy S, Wijaya L, Chan L, et al. Are migrant workers in Singapore receiving adequate healthcare? A survey of doctors working in public tertiary healthcare institutions. Singapore Med J. 2019. Available from: http://www.smj.org.sg/sites/default/files/OA-2019-038-epub.pdf. [cited 31 Aug 2020]. 10.11622/smedj.2019101 PMC7930308 31489436

[40] Ministry of Manpower Singapore. Medical Insurance Requirements for Migrant Workers. Available from: https://www.mom.gov.sg/passes-and-permits/work-permit-for-foreign-worker/sector-specific-rules/medical-insurance. [cited 24 Jun 2023].

[41] Attorney-General's Chambers. Employment of Foreign Manpower (Work Passes) Regulations 2012 - Singapore Statutes Online [Internet]. Singapore. Available from: https://sso.agc.gov.sg:5443/SL/EFMA1990-S569-2012. [cited 7 Sep 2020].

[42] Sadarangani SP, Lim PL, Vasoo S (2017). Infectious diseases and migrant worker health in Singapore: a receiving country’s perspective. J Travel Med.

[43] Ministry of Manpower Singapore. Process Sector: Work Permit Requirements. Available from: https://www.mom.gov.sg/passes-and-permits/work-permit-for-foreign-worker/sector-specific-rules/process-sector-requirements. [cited 19 Jun 2023].

[44] Ministry of Manpower Singapore. Construction Sector: Work Permit Requirements. Available from: https://www.mom.gov.sg/passes-and-permits/work-permit-for-foreign-worker/sector-specific-rules/construction-sector-requirements. [cited 19 Jun 2023].

[45] Ministry of Manpower Singapore. Marine Shipyard Sector: Work Permit Requirements. Available from: https://www.mom.gov.sg/passes-and-permits/work-permit-for-foreign-worker/sector-specific-rules/marine-sector-requirements. [cited 19 Jun 2023].

[46] Dutta M, Kaur-Gill S. Precarities of migrant work in Singapore: Migration, (Im)mobility, and Neoliberal Governmentality. Int J Commun. 2018;1(12):4066–84.

[47] Harrigan N, Koh CY. Vital yet vulnerable: Mental and emotional health of South Asian migrant Workers in Singapore. Res Collection School Soc Sci. 2015;1–51.

[48] Baey G, Yeoh BSA (2018). “The lottery of my life”: migration trajectories and the production of precarity among Bangladeshi migrant workers in Singapore’s construction industry. Asian Pac Migr J.

[49] Ang JW, Chia C, Koh CJ, Chua BWB, Narayanaswamy S, Wijaya L (2017). Healthcare-seeking behaviour, barriers and mental health of non-domestic migrant workers in Singapore. BMJ Glob Health.

[50] Dutta M (2017). Migration and health in the construction industry: culturally centering voices of Bangladeshi workers in Singapore. Int J Environ Res Public Health.

[51] Saw YE, Tan EY, Buvanaswari P, Doshi K, Liu JC (2021). Mental health of international migrant workers amidst large-scale dormitory outbreaks of COVID-19: a population survey in Singapore. J Migration Health.

[52] Glaser B, Strauss A. Discovery of Grounded Theory: Strategies for Qualitative Research. New York: Sage; 1999.

[53] Thomson SB (2010). Grounded theory - sample Size. J Adm Gov.

[54] Tam WJ, Goh WL, Chua J, Legido-Quigley H (2017). 健康是本钱 - Health is my capital: a qualitative study of access to healthcare by Chinese migrants in Singapore. Int J Equity Health.

[55] Corbin JM, Strauss A (1990). Grounded theory research: procedures, canons, and evaluative criteria. Qual Sociol.

[56] Corbin J, Strauss A. Basics of Qualitative Research: Techniques and Procedures for Developing Grounded Theory, 3rd ed. Thousand Oaks: Sage; 2008.

[57] Building and Construction Authority (BCA). Foreign Worker Dormitories. BCA Corp. Available from: https://www1.bca.gov.sg/buildsg/manpower/foreign-worker-dormitories. [cited 16 Aug 2022].

[58] El Alaoui-Faris M, El Alaoui-Faris M, Federico A, Grisold W (2022). Barriers to health for migrants and refugees. Neurology in migrants and refugees.

[59] Chuah FLH, Tan ST, Yeo J, Legido-Quigley H (2018). The health needs and access barriers among refugees and asylum-seekers in Malaysia: a qualitative study. Int J Equity Health.

[60] Loganathan T, Rui D, Ng CW, Pocock NS (2019). Breaking down the barriers: understanding migrant workers’ access to healthcare in Malaysia. PLoS ONE.

[61] Yeoh BSA (2006). Bifurcated labour: the unequal incorporation of transmigrants in Singapore. Tijdschrift voor Econ Soc Geogr.

[62] Zahra M. The legal framework of the sponsorship systems of the Gulf Cooperation Council countries : a comparative examination. 2015; Available from: https://cadmus.eui.eu/handle/1814/37966 [cited 26 Jan 2023].

[63] Fernandez B (2021). Racialised institutional humiliation through the Kafala. J Ethn Migr Stud.

[64] Kanchana R, Chowdhury M, Irudaya Rajan S (2018). Is the Kafala tradition to blame for the exploitative work conditions in the Arab-Gulf countries?. South Asian Migration in the Gulf: Causes and Consequences.

[65] Gardner A. Engulfed: Indian Guest Workers, Bahraini Citizens and the Structural Violence of the Kafala System. The deportation regime : sovereignty, space, and the freedom of movement. 2010; Available from: https://soundideas.pugetsound.edu/faculty_pubs/140.

[66] Internationa Labour Organization. Employer-Migrant Worker Relationships in the Middle East: Exploring scope for internal labour market mobility and fair migration. 2017. Available from: http://www.ilo.org/beirut/publications/WCMS_552697/lang--en/index.htm. [cited 26 Jan 2023].

[67] Harrigan NM, Koh CY, Amirrudin A (2017). Threat of deportation as proximal social determinant of mental health amongst migrant workers. J Immigrant Minority Health.

[68] Fillinger T, Harrigan N, Chok S, Amirrudin A, Meyer P, Rajah M (2017). Labour protection for the vulnerable: an evaluation of the salary and injury claims system for migrant workers in Singapore. Res Collect Schl Soc Sci.

[69] Aye LM, Arokiasamy JT, Barua A, Yadav H, Onunkwor OF (2017). Prevalence and determinants of poor sleep quality among Myanmar migrant workers in Malaysia: a cross-sectional study. J Adv Med Med Res.

[70] Ghasemi SR, Khezeli M, Rajabi-Gilan N, Koulani M, Moloudi-Safa N, Hemati A (2020). Sleep quality and health-related quality of life in workers of Kermanshah Industrial Town: a correlation study. Indian J Occup Environ Med.

[71] Hargreaves S, Rustage K, Nellums LB, McAlpine A, Pocock N, Devakumar D (2019). Occupational health outcomes among international migrant workers: a systematic review and meta-analysis. Lancet Glob Health.

[72] Songkham W, Deeluea J, Suksatit B, Chaiard J (2019). Sleep quality among industrial workers: related factors and impact. J Health Res.

[73] Ministry of Manpower Singapore. Workplace Safety and Health Report 2020. Available from: https://www.mom.gov.sg/-/media/mom/documents/press-releases/2021/0319-annex-a---workplace-safety-and-health-report-2020.pdf. [cited 27 Mar 2021].

[74] Cho HS, Kim YW, Park HW, Lee KH, Jeong BG, Kang YS (2013). The relationship between depressive symptoms among female workers and job stress and sleep quality. Ann Occup Environ Med.

[75] Ministry of Health Singapore. Updates on COVID-19 (Coronavirus Disease 2019) Local Situation. Available from: https://www.moh.gov.sg/covid-19. [cited 30 May 2020].

[76] Lee W, Neo A, Tan S, Cook AR, Wong ML, Tan J (2014). Health-seeking behaviour of male foreign migrant workers living in a dormitory in Singapore. BMC Health Serv Res.

[77] Long Q, Li Y, Wang Y, Yue Y, Tang C, Tang S (2008). Barriers to accessing TB diagnosis for rural-to-urban migrants with chronic cough in Chongqing, China: a mixed methods study. BMC Health Serv Res.

[78] Hong Y, Li X, Stanton B, Lin D, Fang X, Rong M (2006). Too costly to be Ill: health care access and health seeking behaviors among rural-to-urban migrants in China. World Health Popul.

[79] Mathew B, Nambiar D (2020). Understanding the experiences of health care-seeking migrants in Delhi: trajectories and challenges. Qual Health Res.

[80] Regmi PR, Dhakal Adhikari S, Aryal N, Wasti SP, van Teijlingen E (2022). Fear, stigma and othering: the impact of COVID-19 rumours on returnee migrants and muslim populations of Nepal. Int J Environ Res Public Health.

[81] Grove NJ, Zwi AB (2006). Our health and theirs: forced migration, othering, and public health. Soc Sci Med.

[82] International Labour Organization. Labour Migration in the Arab States. 2016. Available from: http://www.ilo.org/beirut/areasofwork/labour-migration/WCMS_514910/lang--en/index.htm. [cited 2 Feb 2022].

[83] Nagaraj A. Trafficked, Exploited, Ransomed - Indian Workers in the Gulf Face New Test. Reuters. 2019. Available from: https://www.reuters.com/article/us-india-gulf-labour-idUSKBN1WF03S. [cited 2 Feb 2022].

[84] Willis HA, Sosoo EE, Bernard DL, Neal A, Neblett EW. The Associations between internalized racism, racial identity, and psychological distress. Emerg Adulthood. 2021;9(4):384–400. 10.1177/21676968211005598 PMC8356901 34395061

[85] Ryan L, Sales R, Tilki M, Siara B (2008). Social networks, social support and social capital: the experiences of recent polish migrants in London. Sociology.

[86] Wong DFK, Leung G (2008). The functions of social support in the mental health of male and female migrant workers in China. Health Soc Work.

[87] Boccagni P (2015). Burden, blessing or both? On the mixed role of transnational ties in migrant informal social support. Int Sociol.

[88] Griffin J, Soskolne V (2003). Psychological distress among Thai migrant workers in Israel. Soc Sci Med.

[89] Lawthom R, Kagan C, Baines S, Lo S, Sham S, Mok L (2013). Experiences of forced labour amongst Chinese migrant workers: exploring the context of vulnerability and protection. Int J Work Organisation Emotion.

[90] Ben-Zur H. Coping styles and affect. Int J Stress Manag. 2009;16(2):87–101.

[91] Kim MS, Duda JL (2003). The coping process: cognitive appraisals of stress, coping strategies, and coping effectiveness. Sport Psychol.

[92] Folkman S, Moskowitz JT (2004). Coping: pitfalls and promise. Annu Rev Psychol.

[93] Baey G, Yeoh BSA. Migration and precarious work: negotiating debt, employment, and livelihood strategies amongst Bangladeshi migrant men working in Singapore’s construction industry. 2015; Available from: https://opendocs.ids.ac.uk/opendocs/handle/20.500.12413/14839 [cited 27 Jan 2023].

[94] Rahman M. Bangladeshi migrant workers in Singapore: The view from inside | United Nations iLibrary. Available from: https://www.un-ilibrary.org/content/journals/15644278/20/1/7. [cited 27 Jan 2023].

[95] International Labour Office (2010). International labour migration: a rights-based approach.

[96] Piper N, Rosewarne S, Withers M. Redefining a rights-based approach in the context of temporary labour migration in Asia. UNRISD Working Paper; 2016 Report No.: 2016–11. Available from: https://www.econstor.eu/handle/10419/186109 [cited 26 Jan 2023].

[97] Ministry of Manpower Singapore. New primary healthcare system for migrant workers. Available from: https://www.mom.gov.sg/newsroom/press-releases/2021/1129-new-primary-healthcare-system-for-migrant-workers. [cited 12 Sep 2022].

[98] Ministry of Manpower Singapore. Mandatory primary care plan to cover outpatient costs For CMP or dormitory-residing Work Permit and S Pass holders. Available from: https://www.mom.gov.sg/newsroom/press-releases/2022/0219-mandatory-purchase-of-primary-care-plans-for-certain-migrant-workers. [cited 12 Sep 2022].

[99] Attorney-General's Chambers. Foreign Employee Dormitories Act 2015 - Singapore Statutes Online. Available from: https://sso.agc.gov.sg:5443/Act/FEDA2015. [cited 12 Sep 2022].

[100] Ministry of Manpower Singapore. Expanded Foreign Employee Dormitories Act To License 1,600 Dormitories Under Single Regulatory Framework. Available from: https://www.mom.gov.sg/newsroom/press-releases/2022/0906-expanded-feda. [cited 12 Sep 2022].

