# Supplementary material for: “My young life, finished already?”: a qualitative study of embedded social stressors and their effects on mental health of low-wage male migrant workers in Singapore
Source: Global Health. 2023 Jul 8;19:47. doi: 10.1186/s12992-023-00946-5 (PMC10329802; doi:10.1186/s12992-023-00946-5)
Supplement: Supplementary file 1 — Additional file 1. Semi-Structured Interview Guide. [file 12992_2023_946_MOESM1_ESM.docx]

**Additional File 1: Semi-Structured Interview Guide**

**I. Living Experiences**

*First of all, I’d like to hear about your experiences with living in dormitories.*

1. How do you like your dormitory like very bad, bad, good or very good? What do you like and don’t like about your dormitory?

[Probe: Refer to MOM policy on what is acceptable accommodation and focus on the following conditions. They are probes, so do not say each term directly. They might not be able to understand. The list is for facilitation]

- - Over-crowd
  - Structural and fire safety
  - Proper drainage and sanitation
  - Hygiene and in-/out-door pollution
  - Adequate water supply and pressure
  - Adequate ventilation
  - Adequate toilet and bathing facilities
  - Secure storage of personal belongings
  1. Is there any particular concern and problem in your dormitory? How did you solve it out?

1. Overall, what do you do to maintain and improve living condition? Are you able to make changes to improve your living conditions? Do you make any rule in the dormitory?

[Probe: collaborative efforts]

1. How easy or difficult to contact the authority to make any adjustments or improvements? Have you tried to contact? What was the result?

[Probe: Try to get the experiences – facts and share the cases with others. Other migrant workers might

- 1. Was there improvement in the dormitory? If so, in what aspect?

**II. Individual and Collective Social Capital**

*Thanks for sharing your experiences and opinions on dormitory. Now, I'd like to hear about your social relationships with others and your social worlds other than workplaces.*

1. When you have spare time what do you usually do? Who do you usually have dinner with?

1. Do you have someone you can trust? Who are they?
2. Do you have someone you can get help and support?
   1. When do you feel stressed and sad?
   2. When you are in need of financial support?
3. When you do not feel well and sick, do you have someone to take care of you?

[Probe: any specific help with medication, referral, company, etc.]

1. Have you ever participated in any community events?

[Probe: soccer, cultural, leisure events, etc.]

[Probe: degree of engagement, role in the organization of the events, volunteer work]

- 1. How did you like the events?

- 1. Any religious activities?

[Probe: see whether there are any health promotion messages from the events]

- 1. What other events would you like to have in the community?

1. Were there any conflicts in your dormitory? If so, how was it resolved?

[Probe: Note! The main purpose of the question is to find out the decision-making process and to identify the role of social networks to address problems. The question does not aim to “document and report” the problem.]

1. Besides the places of your work and living, what are your opinions on the places and opportunities for migrant workers to socialize and participate in personal development in Singapore, like the recreation centre, vocational training, religious institutions, and clinical and legal organizations? Do you think there are many places for migrant workers in Singapore? What are they like?

[Probe: This question might be difficult to follow up. The purpose is to explore the sense of community attachment. Do they see any existing stable networks for their life? If so, what the roles of it?]

[Probe: Transnational community, linkage between home and Singapore]

- 1. In what aspect do they consider the changes or hopes?

**III. Health Promotion**

1. Overall, how do you rate your health? Good or bad? Do you feel that you need medical care?

[Probe: health literacy, perception of health, perceived susceptibility]

[Probe: Yes, most of health concerns would be related to work safety and injuries. Try to get health issues beyond occupational health model.]

1. What do you do to stay healthy, for example eating, exercise, and sleeping well?
   1. If you smoke, have you ever attempted to quit?

[Probe: social cultural context of smoking and seeking help]

- 1. *If you drink, how does it affect daily life and working?*

*[Please: Be mindful when this question is asked – the Bangladeshis are prohibited to drink (Muslim)]*

[Probe: social cultural context of drinking and seeking help]

1. Where do you receive the information on health?

[Probe: source of information from informal and formal networks, such as peers, internet, NGOs, company, or MOM formal training]

1. When you need medical help and look for a medical service, what difficulties do you usually have?

[Probe: financial hardship, employer’s permission, lack of information]

1. What is your experience of using medical services like? How about the last time you used medical services?

[Probe: try to distinguish and compare the services between HealthServe and other clinic settings]

[Probe: And compare to medical services in home country]

1. What difficulties did you have when you receive medical service?

[Probe: language, cultural competency, confidentiality and privacy, perceived stigma, etc; any service provided by healthcare settings to alleviate the difficulties]

[Probe: We can explore social care and legal help in relation to occupational injury]

1. Can you say one or two things that you like the most about the services and tell me why?
   1. For this time, can you say one or two things that you don’t like about and tell me why?

[Probe: For these two questions, try to get the answers from all of them, and invite all to say them quickly. We will move to the last question]

[Probe: If time is running short, skip this question to the next, last question]

**IV. Suggestions for Improvements**

*We are getting to the end of the discussion. We talked about your life and health. Over the discussion, you might have thought about something more. Before we end, I’d like to hear about your opinions if you have.*

1. Do you have further suggestions to improve the dormitory life and healthcare service?

[Probe: We have covered these questions previously. This is a kind of exit question to see if there are any to add. Tailored to bring about a sense of ownership from them]

**V. Debriefing**

*OK. We had a good discussion. Thank you for all of your participation and sharing your views and experience.*

1. There are several topics that we have touched upon. Let me identify some key discussion points we have come up with today.

I'm not looking for further discussion but am checking with your feelings towards what we have discussed. I welcome your clarification if I have any misunderstandings. Do you have any points you want to add?
